# Antifungal activity of nisin against clinical isolates of azole-resistant *Candida tropicalis*

**DOI:** 10.3389/fmicb.2024.1383953

**Published:** 2024-05-07

**Authors:** Shuo Gao, Yueyue Ji, Shilan Xu, Jia Jia, Baiyuan Fan, Yan Zhang, Han Shen, Wanqing Zhou

**Affiliations:** Department of Laboratory Medicine, Nanjing Drum Tower Hospital, Affiliated Hospital of Medical School, Nanjing University, Nanjing, Jiangsu, China

**Keywords:** *Candida tropicalis*, nisin, antifungal activity, biofilm, azole resistance

## Abstract

The rapid emergence of invasive infections caused by azole-resistant *Candida tropicalis* has become a public health concern, and there is an urgent need for alternative treatment strategies. Studies have demonstrated the antibacterial effects of nisin, a well-known peptide naturally produced by *Lactococcus lactis* subsp. *lactis*. However, there is scant information about the antifungal effect of nisin against *C. tropicalis*. The present study aims to investigate the *in vitro* antifungal activity of nisin against clinical isolates of azole-resistant *C. tropicalis* strains, as well as its inhibitory effect on biofilm formation. A total of 35 *C. tropicalis* strains isolated from patients with invasive fungal infections were divided into the azole-resistant group and the azole-sensitive group, containing 21 and 14 strains, respectively. The relative expression levels of the *ERG11* and *UPC2* genes in the azole-resistant group were higher than those in the azole-sensitive group (*p* < 0.0001), while no significant differences were observed in the expression levels of the *MDR1* and *CDR1* genes. The minimum inhibitory concentration of nisin against *C. tropicalis* ranged from 2 to 8 μg/mL. Nisin treatment inhibited the growth of azole-resistant *C. tropicalis,* with over a four-fold reduction in OD_600 nm_ values observed at the 8-h time point, while it promoted the transition of *C. tropicalis* from the spore phase to the hyphal phase, as observed on cryo-scanning electron microscopy. The results of biofilm quantification using crystal violet staining indicated a significant decrease in OD_570 nm_ values in the nisin-treated group compared to the controls (*p* < 0.0001). Among the 21 azole-resistant *C. tropicalis* strains, the biofilm formation was inhibited in 17 strains (17/21, 81%), and more than 85% inhibition of biofilm formation was observed in the representative strains. With regard to the molecular mechanisms, the expression of the *BCR1* and *UPC2* genes in the azole-resistant strains was down-regulated on nisin treatment (*p* < 0.05). In conclusion, we demonstrated, for the first time, that nisin has antifungal activity and significant anti-biofilm activity against clinical isolates of azole-resistant *C. tropicalis* strains. Based on the findings, nisin could be a promising alternative antifungal agent for combating azole-resistant *C. tropicalis* infections.

## Introduction

Fungal infections have become a significant global concern. Among the known fungal pathogens, *Candida tropicalis*, an important opportunistic species, is associated with both superficial and systemic infections, has a higher mortality rate (41%) than other *Candida* species (Andes et al., 2012; de Souza et al., 2023). In 2022, the World Health Organization released its first-ever list of fungal priority pathogens, classifying *C. tropicalis* as a “high priority” fungal pathogen of considerable importance (World Health Organization, 2022). In recent years, the prevalence of drug-resistant *C. tropicalis* has increased, making it a serious-public health threat and a challenge to the management of invasive fungal diseases (Lima et al., 2022).

The most common antifungal drugs used against *Candida* infections were polyenes, fluoropyrimidines, echinocandins, and azoles (de Oliveira Santos et al., 2018). The widespread overuse of fungistatic azoles has led to the emergence of azoles-resistant *C. tropicalis* through various mechanisms, including modification and/or overexpression of the drug target, upregulation of drug-efflux pumps, and compensatory alterations within the ergosterol biosynthesis pathway (Revie et al., 2018). Thus, the clinical usefulness of antifungals is hampered by the undesirable side effects and the emergence of resistance. Additionally, biofilm formation by *C. tropicalis* has enhanced its ability to adhere to epithelial and endothelial cells, and led to an increase in its antibiotic resistance (Queiroz et al., 2023). Therefore, the severity of invasive infections caused by azole-resistant *C. tropicalis* is of the utmost clinical concern, and there is an urgent need for novel strategies to combat these life-threatening infections. These developments have generated greater interest in research on new antifungal drugs with multiple synthetic and natural molecules.

Antimicrobial peptides have emerged as a promising alternative for combating a diverse range of microbial pathogens. Nisin, a 34-amino acid pentacyclic peptide, is one of the oldest known antimicrobial compounds that was first discovered as a possible food-preserving agent in the food industry in 1928 (Rogers and Whittier, 1928). This inhibitory peptide is naturally produced by *Lactococcus lactis* subsp. *lactis* and was approved by the US Food and Drug Agency in 1988, and it generally recognized as safe (de Arauz et al., 2009). Twelve natural variants of nisin have been reported so far, and several bioengineered variants have been developed for various biological applications (Chan et al., 2023). Currently, the focus of research on nisin is shifting from food preservation to its therapeutic use for the treatment of bacterial infections. Nisin exhibits potent activity against Gram-positive bacterial pathogens (Field et al., 2010; Reiners et al., 2020), by causing the formation of pores in the cytoplasmic membrane, disrupting the proton motive force and pH balance, and eventually causing cell death. Another proposed mode of action of nisin is the inhibition of cell wall biosynthesis by binding lipid II, which inhibits cell wall biosynthesis (Field et al., 2008). However, the activity of nisin is substantially weaker against Gram-negative bacteria than against Gram-positive bacteria (Field et al., 2012; Zhou et al., 2023a). While little is known about the antimicrobial activity of nisin against fungi and viruses, a few studies have shown that nisin Z can inhibit the adhesion, growth, and morphological transformation of *C. albicans* (Le Lay et al., 2008; Akerey et al., 2009). However, there are few reports on the antifungal activity of nisin against *C. tropicalis*.

In this study, we have explored the antifungal activity of nisin against clinically isolated azole-resistant *C. tropicalis* and investigated its impact on biofilm formation and the expression of related genes, in order to provide important information for the potential application of nisin as an alternative antifungal therapeutic strategy.

## Materials and methods

### Fungal strains

Fifty-six *Candida* strains including 35 *C. tropicalis* and 21 *C. albicans* strains, isolated from sterile body fluids and blood specimens from patients admitted to Nanjing Drum Tower Hospital between 2013 and 2022 were included. All the strains were identified using the VITEK 2 system (bioMérieux, France) and matrix-assisted laser desorption ionization-time of flight mass spectrometry (MALDI-TOF MS) (bioMérieux, France). Prior to the experiments, *Candida* strains were inoculated on Sabouraud dextrose agar and incubated at 35°C for at least 24 h. *Candida parapsilosis* ATCC 22019 and *Candida krusei* ATCC 6258 were used as quality control strains for antifungal susceptibility testing.

### Antifungal susceptibility testing

Sensititre YeastOne™ YO10 (ThermoFisher Scientific, Cleveland, OH, USA) was utilized for antifungal susceptibility analysis following the manufacturer’s instructions. Current clinical breakpoints or epidemiological cut-off values were employed for interpretation of the susceptibility results (Clinical and Laboratory Standards Institute, 2022). The MICs of nisin (MCE, USA) against 35 clinical isolates of *C. tropicalis* and 21 isolates of *C. albicans* were determined using the broth microdilution method in accordance with the CLSI guidelines (M27-Ed4) (Clinical and Laboratory Standards Institute, 2017). Briefly, nisin was dissolved in 0.02 mol/L HCl and prepared at a concentration of 2048 μg/mL for storage (Santos et al., 2019). RPMI-1640, buffered to pH 7 with 3-[N-morpholino] propanesulfonic acid, was used as the growth medium, and the fungal solutions were incubated in a U-shaped 96-well sterile microtiter plate for 24 h at 35°C. The MIC was determined as the lowest concentration resulting in 50% inhibition of growth compared to the growth of the control. *C. parapsilosis* ATCC 22019 and *C. krusei* ATCC 6258 were included with each assessment as quality controls.

### Growth inhibition curves

*Candida* strains were grown in yeast extract peptone dextrose broth (YPD, containing 1% yeast extract, 2% peptone, and 2% glucose) at 35°C for 18 h. The fungal inoculum standardized until a cell concentration of 10^5^ CFU/mL was reached and then treated with nisin, which was diluted to concentration of 1,024 μg/mL, 512 μg/mL, and 256 μg/mL in the final incubation, with shaking at 180 rpm. HCl at concentrations of 0.01 mol/L, 0.005 mol/L and 0.0025 mol/L were set up as reagent controls. Cells cultivated only with YPD broth was set as the blank control. Subsequently, absorbance was measured at OD_600 nm_ using a MB-580 microplate reader (HEALES, China) at 0, 2, 4, 8, 12, 16, and 24 h, respectively. All assays were performed in triplicate.

### Cryo-scanning electron microscope

A cryo-scanning electron microscope (Hitachi Regulus 8,100, Japan) was utilized to observe the effect of nisin on *C. tropicalis*. The cells were cultured in both drug-free medium and a medium containing nisin (8 μg/mL) for 24 h. The process involved loading the fungal sample onto the sample holder, mounting the sample holder onto the sample transfer rod device, and then inserting the device into solid nitrogen at −210°C for approximately 30 s for pre-cooling and cryofixation. The sample was then transferred under vacuum to the cryo-preparation chamber at −90°C, where it was sublimated for 2 min at −95°C to remove water from the sample and coated with a conductor by spraying with platinum for 50 s. Once the cryogenic sample preparation was completed, the sample could be transferred to the cold stage of the scanning electron microscope, with the temperature set at −175°C, for observation and photography.

### Biofilm inhibition

The biofilm formation ability of *C. tropicalis* and *C. albicans* strains was evaluated using the microtiter plate method, as described previously (Zhou et al., 2023b). The strains were first grown overnight on a YPD agar plate, adjusted to a concentration of 0.5 McFarland, and then seeded at a concentration of 10^6^ CFU/mL in YPD broth. Subsequently, diluted broth containing nisin at 1/2 MIC or a corresponding concentration of HCl was added, with a final volume of 200 μL per well, and the microtiter plate was incubated at 35°C for 24 h. After incubation, the solution was discarded, and the plate was washed with sterile phosphate-buffered saline three times and dried. The plate was then stained with 100 μL of 0.5% crystal violet (Beyotime Biotechnology, China) for 15 min and washed with sterile PBS three times. Following this, 200 μL of absolute ethanol was added, and the absorbance was measured at OD_570 nm_ using a microplate reader (Zhou et al., 2022). Each assay was performed with three replicates for each strain. Sterile YPD broth served as a blank control, while *S. epidermidis* ATCC 12228 and ATCC 35984 were used as the negative and positive controls, respectively (Solis et al., 2016).

### Quantitative reverse transcription polymerase chain reaction

Quantitative reverse transcription polymerase chain reaction (qRT-PCR) was utilized to assess the expression of the *ERG11*, *MDR1*, *CDR1*, *UPC2,* and *BCR1* genes. RNA extraction was carried out using the RNA Enhancement and FreeZol Reagent (Vazmye, China) according to the manufacturer’s instructions. *C. tropicalis* strains were initially cultured on YPD medium, and this was followed by incubation in YPD broth containing nisin (1/2 MIC) at 35°C with shaking at 180 rpm for 8 h. Subsequently, the culture was collected for RNA extraction. cDNA synthesis was immediately performed for each sample in two steps using a HiScript RT SuperMix qPCR kit (Vazmye, China) based on the manufacturer’s instructions. qPCR was conducted in a 20-μL reaction volume using ChamQ Universal SYBR qPCR Master Mix (Vazmye, China). The reactions commenced with primary denaturation at 95°C for 2 min, which was followed by 40 cycles each of 95°C for 10 s and 60°C for 30 s. *ACT1* was employed as an internal reference. All treatments were carried out in triplicate, and the 2^−ΔΔCt^ method was used to determine the fold changes of the candidate genes.

### Statistical analysis

The data were analyzed using the SPSS statistical software (version 20.0) and GraphPad (Version 9, GraphPad Software, United States). Quantitative data were subjected to the *t*-test or the Mann–Whitney *U*-test. Statistical significance was set at 0.05.

## Results

### Susceptibility of *Candida tropicalis* to antifungal drugs

Antifungal susceptibility testing was conducted on 35 strains of *C. tropicalis*, which were divided into azole-resistant (21 strains) and azole-sensitive (14 strains) groups based on their MIC values for fluconazole, itraconazole, voriconazole, and posaconazole. The MICs were higher in the azole-resistant groups (fluconazole: 128–256 μg/mL, itraconazole: 0.5–16 μg/mL; voriconazole: 4–8 μg/mL; and posaconazole: 0.25–2 μg/mL) than in the azole-sensitive group (fluconazole: ≤ 2 μg/mL; itraconazole: ≤ 0.25 μg/mL; voriconazole: ≤ 0.12 μg/mL; and posaconazole: ≤ 0.12 μg/mL). All the 35 strains were sensitive to amphotericin B, anidulafungin, caspofungin, and micafungin.

### Expression of genes related to azole resistance in *Candida tropicalis*

**Figure 1:**
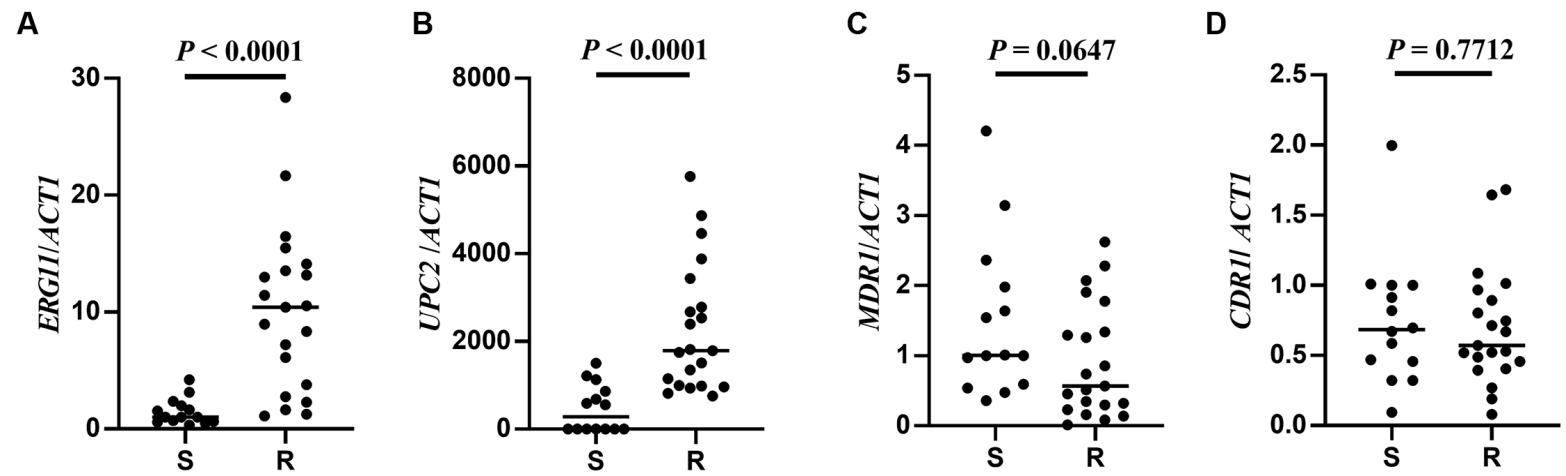
Azole-resistant related genes expression of *C. tropicalis*. Expression levels of resistant-related genes *EGR11* **(A)**, *UPC2* **(B)**, *MDR1* **(C)** and *CDR1* **(D)** in azole-sensitive and azole-resistant groups by qRT-PCR, respectively. S: azoles sensitive group, *n* = 14. R: azoles resistant group, *n* = 21. Mann–Whitney *U* test was used to compared the genes expression between two groups.

In order to investigate the resistance mechanisms of the azole-resistant *C. tropicalis* strains included in our study, the relative expression levels of the *ERG11*, *UPC2*, *MDR1*, and *CDR1* genes in azole-resistant and azole-sensitive strains were evaluated using qRT-PCR. The results indicated that the relative expression levels of the *ERG11* and *UPC2* genes were significantly higher in the azole-resistant group than in the azole-sensitive group (Figures 1A,B, *p*< 0.0001), while no differences were observed for the *MDR1* and *CDR1* genes (Figures 1C,D, *p* > 0.05). These results suggest that overexpression of the *ERG11* and *UPC2* genes may be the main mechanism in the isolated azole-resistant *C. tropicalis* strains investigated here.

### Antifungal activity of nisin against *Candida tropicalis*

The MIC values of nisin were determined for various strains and were found to range between 2 to 8 μg/mL for clinical *C. tropicalis* isolates (MIC_90_ and MIC_50_ were both 4 μg/mL) and 1 to 2 μg/mL for *C. albicans* strains (MIC_90_ and MIC_50_ were both 2 μg/mL). With regard to the controls, the MIC values of nisin against *C. parapsilosis* ATCC 22019 and *C. krusei* ATCC 6258 were 2 μg/mL and 4 μg/mL, respectively, as shown in Table 1.

**Table 1:** MIC values of nisin against fungi strains.

Fungi	Strains Numbers	Nisin (μg/mL)			
		MIC	MIC_90_	MIC_50_	100% inhibition
*C. tropicalis*	35	2–8	4	4	64–512
*C. albicans*	21	1–2	2	2	8–256
*C. parapsilosis* ATCC 22019	1	2	/	/	8
*C. krusei* ATCC 6258	1	4	/	/	16

MIC, minimal inhibitory concentration.

**Figure 2:**
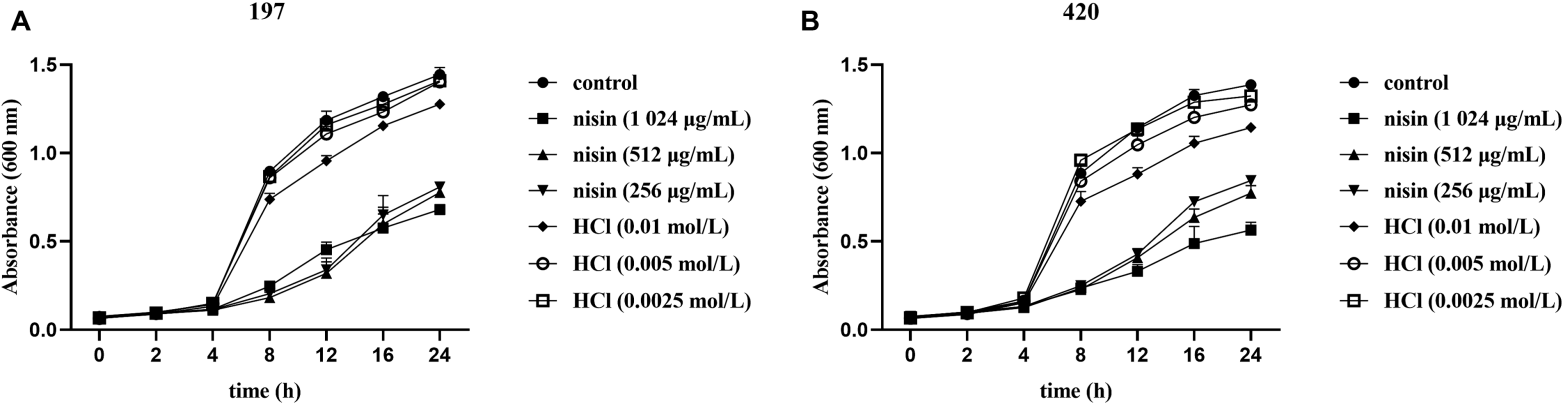
Impact of nisin on the growth of *C. tropicalis* isolates. The growth inhibition curves of *C. tropicalis* no.197 **(A)** and 420 **(B)** were performed. The fungal inoculum was standardized until reaching a cell concentration of 10^5^ CFU/mL, then treated with nisin which was diluted to the concentration of 1,024 μg/mL, 512 μg/mL and 256 μg/mL in the final incubation with shaking at 180 rpm in tubes. Subsequently, growth was measured using absorbance at OD_600 nm_ in YPD medium at times 0, 2, 4, 8, 12, 16, and 24 h in a MB-580 microplate reader (HEALES, China). YPD broth and YPD containing 0.01 mol/L, 0.005 mol/L, 0.0025 mol/L HCl in final concentrations were used as reagent controls. Cells cultivated only with YPD broth was set as the blank control. All assays were performed in triplicate.

*Candida tropicalis* clinical isolates numbered 197 and 420 were randomly selected for nisin inhibition fungal growth curve analysis. The concentration at which nisin completely inhibited fungal growth was 512 μg/mL. Consequently, we selected concentrations of 1,024 μg/mL, 512 μg/mL and 256 μg/mL of nisin for the analysis of growth inhibition curves. After 24 h of growth observation, we assessed the inhibitory effect of the above concentrations of nisin on *C. tropicalis* by measuring the reduction in absorbance at OD_600 nm_. Our results demonstrated that nisin at concentrations of 1,024 μg/mL, 512 μg/mL and 256 μg/mL resulted in a significant decrease in the growth of *C. tropicalis* over 8–24 h (Figures 2A,B). Nisin exhibited dose- and time-dependent inhibition of *C. tropicalis* growth. Specifically, treatment of strain 197 with 512 μg/mL of nisin resulted in a more than four-fold reduction in absorbance at the 8-h time point (0.8611 ± 0.0191 *vs* 0.1813 ± 0.0301, *p* < 0.0001). Moreover, the concentrations of HCl with 0.005 mol/L did not have interference with the viability of fungal growth.

### Effects of nisin on fungal micromorphology

**Figure 3:**
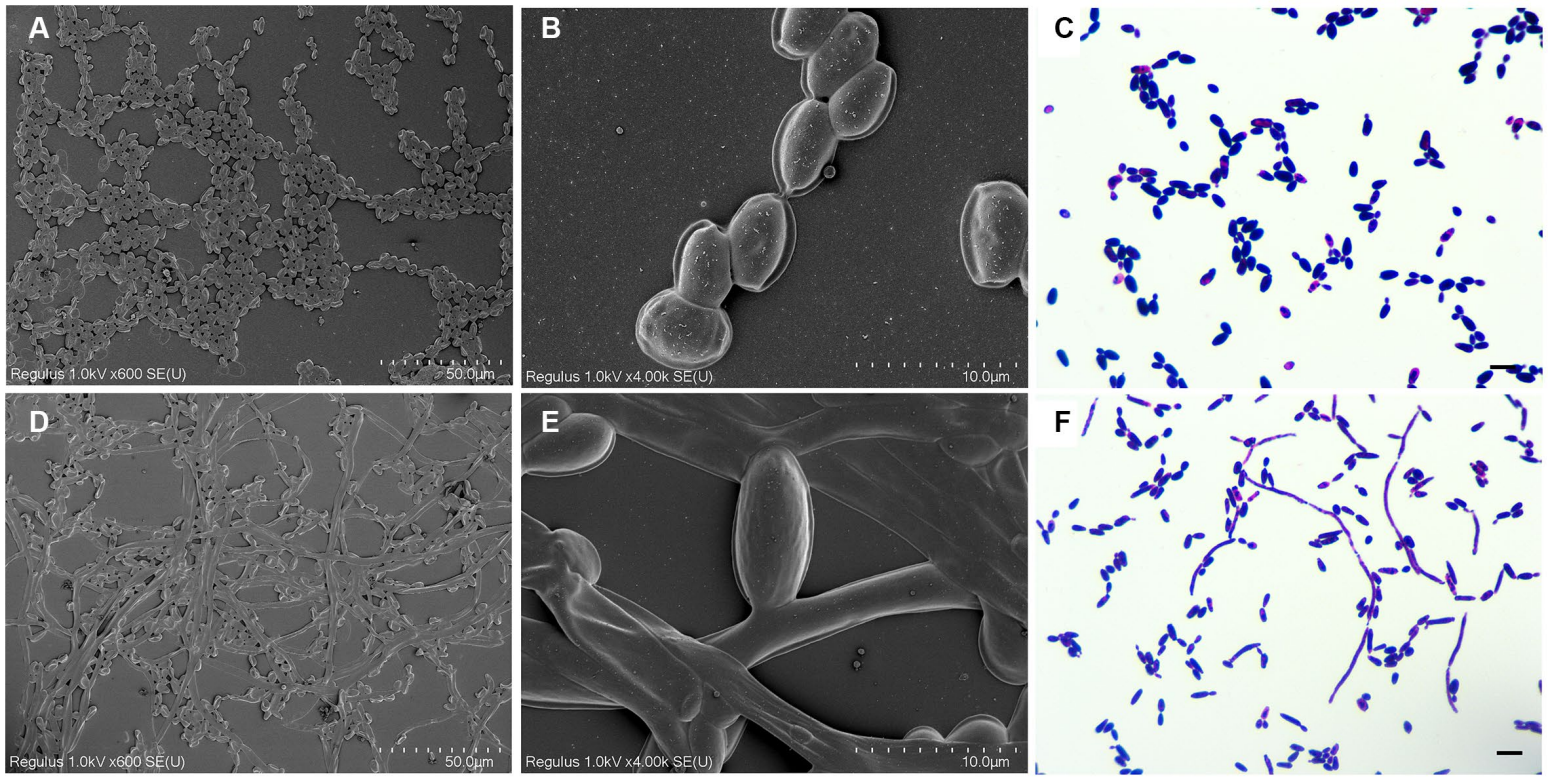
Nisin promoted *C. tropicalis* yeast-hypha transition. The *C. tropicalis* cells were cultured in drug-free medium and medium with nisin (8 μg/mL) for 24 h. Control, cryo-scanning electron microscope 1.0KV × 600 **(A)**. Control, cryo-scanning electron microscope 1.0KV × 4000 **(B)**. Control, Gram staining **(C)**. Nisin, cryo-scanning electron microscope 1.0KV × 600 **(D)**. Nisin, 1.0KV × 4000 **(E)**. Nisin, Gram staining **(F)**. Scale: 10 μm.

To investigate the potential impact of nisin on the morphology of azole-resistant *C. tropicalis*, we employed cryo-scanning electron microscopy and Gram staining. *C. tropicalis* was cultured in both a drug-free medium and a medium containing nisin (8 μg/mL) for 24 h. Our observations using cryo-scanning electron microscopy and Gram staining revealed significant alterations in the morphology of the nisin-treated cells compared to the control cells. Under normal culture conditions, *C. tropicalis* exhibited a typical cellular morphology characterized by a rounded or oval shape with a smooth surface (Figures 3A–C). However, after 24 h of incubation with nisin, we observed apparent transition from the yeast to the hyphal form, as well as the presence of ultrastructural collapse with loss of shape (Figures 3D–F). In addition, vesicle-like structures were found when *C. tropicalis* was cultured in liquid media (Figures 3B,E).

### Inhibitory effect of nisin on biofilm formation and expression of related genes

**Figure 4:**
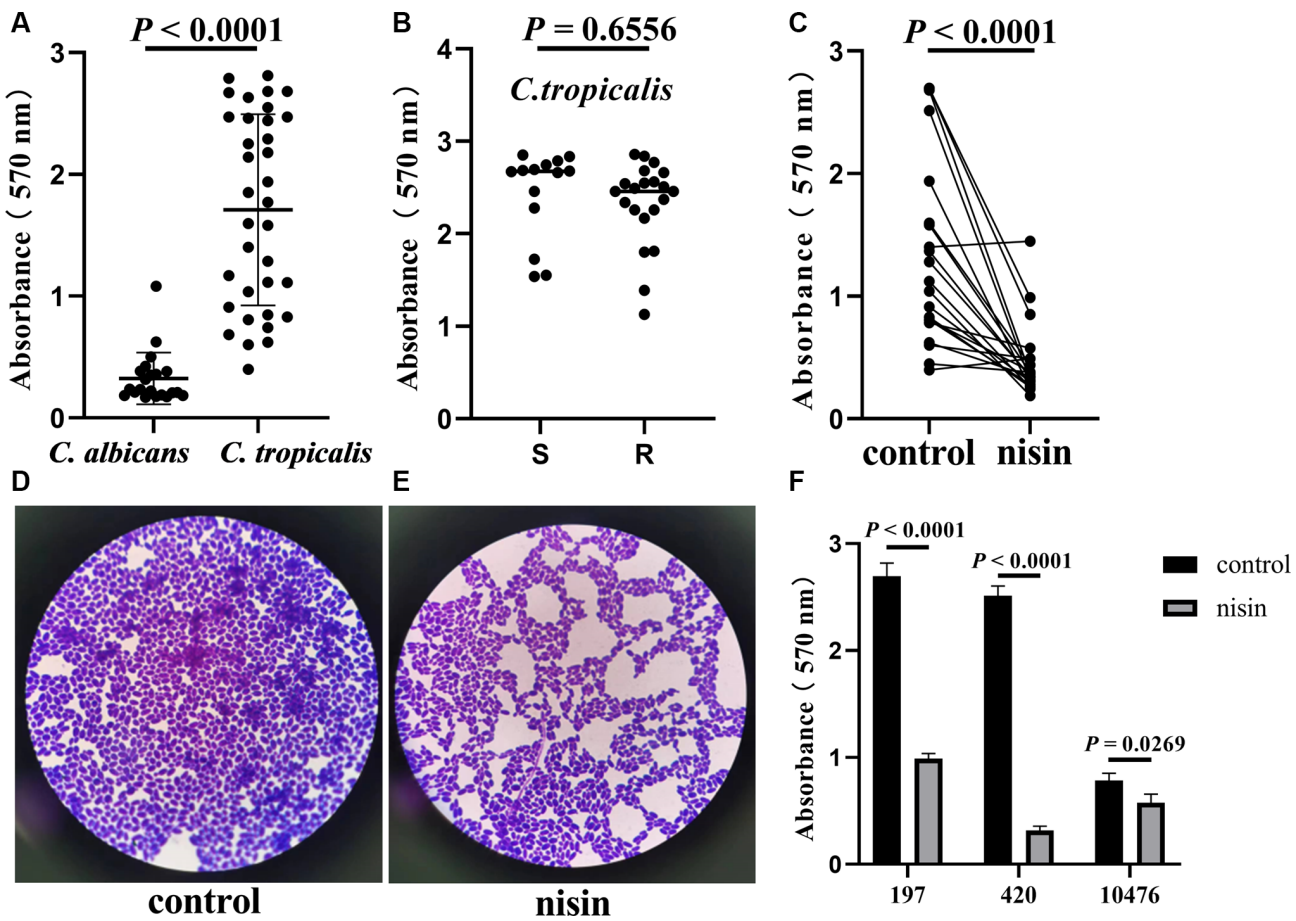
Inhibitory effect of nisin on the *in vitro* biofilm formation of *C. tropicalis*. Absorbance at OD_570 nm_ of *C. albicans* (*n* = 21) and *C. tropicalis* (*n* = 35) strains **(A)**, also azole-sensitive and azole-resistant groups of *C. tropicalis* **(B)**. S: azoles sensitive group, *n* = 14. R: azoles resistant group, *n* = 21. Absorbance at OD_570 nm_ of control and nisin treatment group **(C)**. The crystal violet stain of control **(D)** and nisin treatment group **(E)** under microscope. The inhibition for representative strains with their specific controls **(F)**. The concentration of nisin is 1/2 MIC. Mann–Whitney *U* test was used to compared the inhibitory effect between two groups.

*Candida tropicalis* exhibited a stronger ability to form biofilms than *C. albicans* (Figure 4A). However, there was no significant difference in biofilm formation ability observed between the azole-resistant and azole-sensitive groups of *C. tropicalis* (Figure 4B). However, treatment with nisin at 1/2 MIC for 24 h, resulted in a significant decrease in the OD_570 nm_ value of the 21 azole-resistant *C. tropicalis* strains compared to the controls (Figure 4C, *U* = 40, *p* < 0.0001). Among the 21 azole-resistant *C. tropicalis* strains, the biofilm formation was inhibited in 17 strains (17/21, 81%). The biofilm biomass of *C. tropicalis* was significantly decreased compared to the control (Figures 4D,E), and the rate of inhibition of biofilm formation was more than 85% in strain 420 (Figure 4F). Consequently, our findings indicate that nisin effectively reduces the biofilm production by azole-resistant *C. tropicalis* strains.

**Figure 5:**
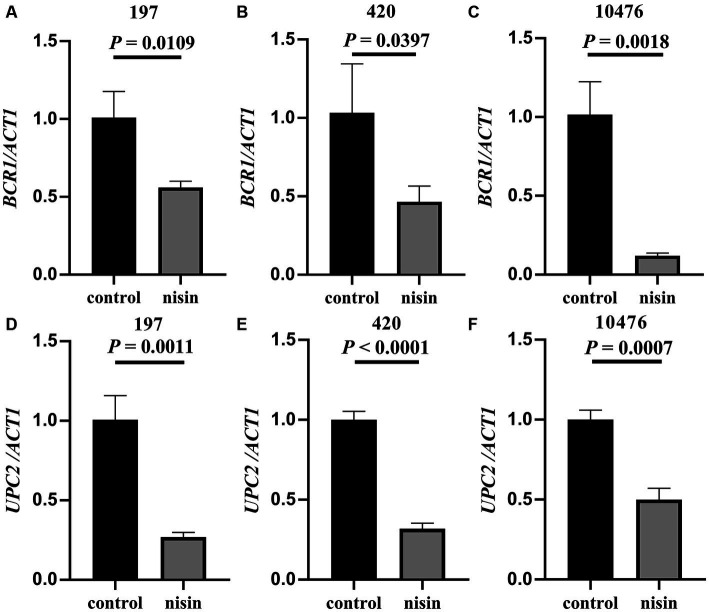
Nisin inhibited the expression of *BCR1* and *UPC2* genes in *C. tropicalis*. Relative mRNA expression levels of *BCR1* **(A–C)** and *UPC2* **(D–F)** in strains of *C. tropicalis* 197, 420 and 10,476 were detected with qRT-PCR. Unpaired *t*-test.

To further investigate these effects of nisin at the gene level, we quantified the difference in the expression of the *BCR1* and *UPC2* genes in three representative *C. tropicalis* strains incubated with or without nisin for 8 h using qRT-PCR. The results demonstrated that the expression level of the *BCR1* gene, a regulator of biofilm generation, was downregulated by nisin (1/2 MIC) in strains 197, 420, and 10,476 (Figures 5A–C, *p* < 0.05). Additionally, the resistance-related gene *UPC2* also exhibited decreased expression after treatment with nisin (1/2 MIC) (Figures 5D–F, *p* < 0.05).

## Discussion

Nisin, a well-studied and widely used lantibiotic, has been shown to have extremely potent against a variety of Gram-positive bacteria, while little is known about the antimicrobial activity of nisin against fungi (Khan et al., 2023). A few studies suggested that nisin may possess antifungal activity on *C. albicans*. Le Lay et al. first investigated the effect of nisin Z on *C. albicans* and demonstrated its ability to inhibit the growth of *C. albicans* strains at concentrations of 500 μg/mL, in a dose- and time-dependent manner (Le Lay et al., 2008). Other studies have suggested that nisin Z may inhibit *C. albicans* adhesion and transition (Akerey et al., 2009). However, there were few reports on the effectiveness of nisin against *C. tropicalis*, which is one of the most important *Candida* species with high rate of mortality and an increasing azole-resistant rates (Wang et al., 2021b). Our study demonstrated, for the first time, that nisin has antifungal activity and significant anti-biofilm activity against clinical isolates of azole-resistant *C. tropicalis* strains.

Some factors that contribute to the azole resistance of *Candida* spp. include mutations and/or overexpression of the following genes: *ERG11* (encodes cytochrome P450 lanosterol 14a-demethy-lase), *UPC2* (encodes the transcription factor of *ERG11* gene), *CDR1* (encodes efflux protein of ATP binding-cassette (ABC) family) and *MDR1* (encodes efflux protein of major facilitator superfamily (MFS) family) (Sasani et al., 2021). In the present study, we observed that clinically isolated azole-resistant *C. tropicalis* exhibited high expression of *ERG11* and *UPC2* genes (as shown in Figures 1A,B). Further, the azole-resistant strains exhibited higher MIC values for fluconazole and voriconazole. These findings are similar to those reported in Wang’s study (Wang et al., 2021a). However, our study did not find any significant difference in the expression levels of *CDR1* and *MDR1* genes between azole-resistant and azole-susceptible *C. tropicalis* isolates. These results imply that the *ERG11* and *UPC2* genes play a role in the molecular mechanisms of azole resistance in *C. tropicalis* isolates.

The discovery of new antifungal drugs for clinical treatment of the resistant strains is urgently needed, and nisin has piqued our interest as a potential safe and effective choice against *C. tropicalis*, especially for drug-resistant strains. In this study, we investigated the efficacy of nisin against *C. tropicalis*, with a primary focus on its ability to control fungal growth. Our findings revealed that concentrations of 2–8 μg/mL of nisin can inhibit the growth of clinical strains of *C. tropicalis*, including azole-resistant strains with MIC_50_ and MIC_90_ both being 4 μg/mL. Compared to clinical *C. tropicalis* strains, the MIC of nisin against *C. albicans* was lower than that against *C. tropicalis*. Furthermore, considering that the concentration at which nisin completely inhibited *C. tropicalis* growth was 512 μg/mL, we used nisin at concentrations of 1,024 μg/mL, 512 μg/mL and 256 μg/mL to perform the fungal growth inhibition experiments. The results showed that nisin at concentrations of 1,024 μg/mL, 512 μg/mL, and 256 μg/mL significantly inhibited fungal growth in dose and time-dependent manner. These results indicate that nisin has potential as an alternative antifungal strategy to target azole-resistant *C. tropicalis*. Recently, nisin-loaded PCL nanoparticles have been suggested as a potential strategy for preventing vaginal candidiasis by inhibiting *C. albicans* growth (de Abreu et al., 2016). Further, the use of nisin as an additive may help reduce the use of antibiotics, minimize side effects, and prevent resistance in clinical scenarios (Chan et al., 2023). Therefore, nanoformulations or combination treatment therapies that include nisin may hold promise for combating fungal infections and entail exciting prospects for future applications.

Nisin exhibits significant antimicrobial activity primarily through electrostatic interactions with bacterial cell wall constituents, such as lipid II, teichoic acid, and polysaccharides. This interaction leads to the formation of stable toroidal pores, which inhibit cell wall biosynthesis and ultimately cause cell death (Bauer and Dicks, 2005). Based on these observations, it is possible that the antifungal mechanism of nisin also relies on disrupting the integrity of fungal cell walls and affecting the transition between yeast and hyphal forms. Accordingly, previous studies have reported that nisin Z-treated *C. albicans* had distorted cell wall surfaces and highly vacuolated cells (Le Lay et al., 2008). Further, it has also been reported that the transition between the yeast and hyphal forms is closely linked to the adaptability and pathogenicity of *Candida* in the host environment (Jain et al., 2008). In the case of *C. albicans*, it has been demonstrated that nisin Z effectively inhibits the transformation of *C. albicans* from blastospores to hyphal forms, thereby disrupting its transition (Le Lay et al., 2008). This differs from our observation on *C. tropicalis* treated with nisin, as shown in Figure 3: *C. tropicalis* treated by nisin showed yeast-hypha transition and exhibited ultrastructural collapse with loss of shape. However, the impact on internal cell damage is unclear and needs to be further investigated. The regulatory mechanisms of filamentation in *C. tropicalis* exhibit both conserved and divergent features (Zhang et al., 2016), so, further research is needed to determine if the regulation of filamentation by nisin is specific to *C. tropicalis*. Additionally, recent research indicated that fungal extracellular vesicles can regulate yeast-to-hypha differentiation in *C. albicans* (Honorato et al., 2022). In our study, vesicle-like structures were also found through observation using cryo-electron microscopy after culturing of *C. tropicalis* in liquid media (Figures 3B,E).

Biofilm formation is one of the main virulence factors in *C. tropicalis*. A recent study has demonstrated that biofilm production in *C. tropicalis* was associated with high mortality rates in patients with candidemia (Vitális et al., 2020). Furthermore, biofilm growth is accompanied by resistance to antifungal drugs, especially azoles, in *C. tropicalis* (Borghi et al., 2016; Cavalheiro and Teixeira, 2018). According to our results, *C. tropicalis* has stronger ability to form biofilms than *C. albicans*, but no differences were found between azole-resistant and azole-sensitive strains. In the clinical environment, the colonization adherence of a pathogen to abiotic surfaces is the first step in biofilms formation on medical devices. Catheter-related blood *C. tropicalis* isolates exhibit a stronger adhesion ability to polystyrene microspheres than that of other sources of *Candida* species (Zuo et al., 2021), and *C. tropicalis* strains isolated from the urinary tract can form biofilms on silicone and latex urinary catheters (Negri et al., 2011). Several studies have shown that nisin has anti-biofilm activity against clinical isolates of *Staphylococcus* and *Pseudomonas aeruginosa* (Field et al., 2016; Chen et al., 2023). Furthermore, nisin Z, in combination with gingival cells, downregulated *C. albicans* adhesion (Akerey et al., 2009). Further, a new study has shown that the curcumin-nisin-poly (L-lactic acid) nanoparticle could serve as an excellent orthodontic acrylic resin additive against *S. mutans* and *C. albicans* biofilms (Pourhajibagher et al., 2022). In our results, as shown in Figures 4C–F, we found for the first time that nisin can significantly inhibit biofilm formation of azole-resistant *C. tropicalis* strains, 81% inhibition of biofilm was observed (17/21), while more than 85% inhibition of biofilm formation was observed in the representative strains. Thus, due to its effectiveness and low toxicity, nisin has potential value in inhibiting the colonization of *C. tropicalis* in the clinical environment.

The formation of biofilms in *Candida* species is regulated by a heterogeneous gene network involved in adhesion, extracellular matrix, and filamentation (de Souza et al., 2023). Nisin used independently or in combination with other antimicrobials has been found to reduce adhesion to polystyrene and the expression of genes related to biofilm formation (Mathur et al., 2018). In *C. tropicalis*, the transcription factor Bcr1 regulates the expression of adhesin-associated genes such as *CtrALS1*, *CtrALS3*, and *HWP1* (Zhang et al., 2016; Staniszewska, 2020). Accordingly, our results demonstrate that nisin can decrease the expression of the *BCR1* gene, which may contribute to the regulation of biofilms and cell walls. Sasani et al. (2021) showed an increase in the expression of both the *ERG11* and *UPC2* genes in fluconazole-treated biofilms of *C. tropicalis*. Here, we found that nisin can reduce the expression of the *UPC2* gene in azole-resistant *C. tropicalis* strains (Figure 5). As a transcription factor of *ERG11* genes, the *UPC2* gene plays an important role in the mechanism of azole resistance in *C. tropicalis*. However, whether the downregulation of *UPC2* by nisin implies that combining nisin with azole drugs could have a synergistic clinical effect needs further investigation. By elucidating the inhibitory effects of nisin on both biofilm formation and gene expression in *C. tropicalis*, this study provides valuable insights into the potential application of nisin as a therapeutic agent against biofilm-related infections caused by azole-resistant strains. Further research is warranted to explore the underlying mechanisms and optimize the utilization of nisin in clinical settings.

## Conclusion

In conclusion, our study explored, for the first time, the antifungal activity of nisin against clinical isolates of azole-resistant *C. tropicalis*. We found that nisin was able to inhibit the growth of azole-resistant *C. tropicalis* and prevent biofilm formation; further, the mechanistic experiments showed that these effects involve the downregulation of the *BCR1* gene. We also observed that nisin treatment can downregulate *UPC2* gene expression, and this suggests its potential application in combating resistant strains or in combination with other antifungal drugs. One limitation of our study was the insufficient number of *C. tropicalis* strains, which will be increased in subsequent research. In addition, there were technical limitations on morphological research. Therefore, further in-depth observation and study are needed to explore the finer structural changes in *C. tropicalis* after the action of nisin. Although nisin did not inhibit filamentation in our study, further investigation to understand the possible molecular mechanisms behind this yeast-to-hyphae transition is necessary. Our findings contribute new evidence to the field of antifungal research on nisin. Still, the antifungal activity of nisin remains understudied and warrants further study, as it may emerge as a promising candidate for future strategies in treating clinical azole-resistant *C. tropicalis* infections.

## Data availability statement

The original contributions presented in the study are included in the article/supplementary material, further inquiries can be directed to the corresponding author.

## Author contributions

SG: Data curation, Writing – original draft, Conceptualization. YJ: Data curation, Software, Writing – review & editing. SX: Data curation, Methodology, Writing – review & editing. JJ: Formal analysis, Investigation, Writing – review & editing. BF: Data curation, Investigation, Writing – review & editing. YZ: Formal analysis, Methodology, Writing – review & editing. HS: Resources, Writing – review & editing. WZ: Funding acquisition, Project administration, Writing – original draft, Writing – review & editing.
